# Exposure to Radiofrequency Radiation Emitted from Common Mobile Phone Jammers Alters the Pattern of Muscle Contractions: an Animal Model Study

**Published:** 2015-09-01

**Authors:** A. Rafati, S. Rahimi, A. Talebi, A. Soleimani, M. Haghani, S. M. J. Mortazavi

**Affiliations:** 1 Physiology Department, School of Medicine, Shiraz University of Medical Sciences, Shiraz, Iran; 2 Medical Physics and Medical Engineering Department, School of Medicine, Shiraz University of Medical Sciences, Shiraz, Iran; 3 Department of Epidemiology, school of health, Tabriz university of medical science, Tabriz, Iran; 4 Ionizing and Non-ionizing Radiation Protection Research Center (INIRPRC), Shiraz University of Medical Sciences, Shiraz, Iran

**Keywords:** Non-Ionizing Radiation, Radiofrequency (RF), Electromagnetic Fields, GSM Mobile Phone, Muscle Contractions, Frog

## Abstract

**Introduction:**

The rapid growth of wireless communication technologies has caused public concerns regarding the biological effects of electromagnetic radiations on human health. Some early reports indicated a wide variety of non-thermal effects of electromagnetic radiation on amphibians such as the alterations of the pattern of muscle extractions. This study is aimed at investigating the effects of exposure to radiofrequency (RF) radiation emitted from mobile phone jammers on the pulse height of contractions, the time interval between two subsequent contractions and the latency period of frog’s isolated gastrocnemius muscle after stimulation with single square pulses of 1V (1 Hz).

**Materials and Methods:**

Frogs were kept in plastic containers in a room. Animals in the jammer group were exposed to radiofrequency (RF) radiation emitted from a common Jammer at a distance of 1m from the jammer’s antenna for 2 hours while the control frogs were only sham exposed. Then animals were sacrificed and isolated gastrocnemius muscles were exposed to on/off jammer radiation for 3 subsequent 10 minute intervals. Isolated gastrocnemius muscles were attached to the force transducer with a string. Using a PowerLab device (26-T), the pattern of muscular contractions was monitored after applying single square pulses of 1V (1 Hz) as stimuli.

**Results:**

The findings of this study showed that the pulse height of muscle contractions could not be affected by the exposure to electromagnetic fields. However, the latency period was effectively altered in RF-exposed samples. However, none of the experiments could show an alteration in the time interval between two subsequent contractions after exposure to electromagnetic fields.

**Conclusion:**

These findings support early reports which indicated a wide variety of non-thermal effects of electromagnetic radiation on amphibians including the effects on the pattern of muscle extractions.

## Introduction

The fast-growing telecommunication and wireless technologies and more dependence to these communication devices have led to higher levels of exposure to electromagnetic radiations emitted by different sources such as cellular phones and their base stations. Mobile phone jammers are devices that emit radiofrequency radiation in the same frequencies that mobile phones operate to disable signaling in places where silence is valued or where information quarantine measures are required such as examination halls, prisons, or military centers. Rapid growth of wireless communication technologies, has led to public concern regarding the biological effects of electromagnetic radiations on human health. Understanding the effects of exposure of humans or higher animals to electromagnetic fields (EMFs) is clearly associated with the understanding of the targets of these fields in exposed cells and tissues. However, it is still unclear what these targets are and how they may cause complex biological responses to very low-energy non-ionizing radiations. It is puzzling that low-energy photons (in energy ranges which usually cannot individually alter the chemistry of the cell) can lead to non-thermal effects in the irradiated cells [1]. The function of brain and nervous systems is based on using electrical signals. Hence, brain and nervous systems can be particularly vulnerable to low frequency EMFs and the electric fields and currents which are induced during exposure. It is worth mentioning that some of the studies performed on animal models have shown that the bioeffects of exposure to electromagnetic fields can be mediated by the nervous system. These studies have revealed that several biochemical changes in nervous systems (alterations in water, oxygen, calcium and some regulatory peptides such as serotonin and histamine) are associated with exposure to electromagnetic fields.

Over the past years, our laboratory has focused on studying the health effects of exposure of laboratory animals and humans to some common and/or occupational sources of electromagnetic fields such as mobile phones [2-9] and their base stations [10], mobile phone jammers [11], laptop computers [12], radars [3], dentistry cavitrons [13] and MRI [14, 15].  This study is aimed at investigating the effects of exposure to radiofrequency radiation emitted by a mobile jammer on the pulse height of contractions, the time interval between two subsequent contractions and the latency period of frog’s isolated gastrocnemius muscle after stimulation with single square pulses of 1V (1 Hz)

## Material and Methods

### Animals

Frogs of both sexes (20-30 g) were obtained from Animal Lab of the Physiology Department, SUMS. Animals were kept in plastic containers in a room (20 ± 1 °C) for one week before the experiments. The tap water in the plastic containers was changed 2 times a week.

### Exposure

Control frogs were kept in special cages during the sham exposure phases, while the jammer group was exposed to radiofrequency (RF) radiation emitted from a common Jammer at a distance of 1m from the jammer’s antenna for 2 hours. Isolated gastrocnemius muscles, in the next stage, were exposed to on/off jammer radiation for 3 subsequent 10 minute intervals.

### Experiment Setup

Frogs were double pithed using a needle. Then the skin over the gastrocnemius muscle and its distal tendon was removed. Isolated gastrocnemius muscles were attached to the force transducer with a string. Nerve and muscle stimulations were performed separately. Using a PowerLab device (26-T), the pattern of muscular contractions was monitored after applying single square pulses of 1V (1 Hz) as stimuli. The pulse height of contractions, the time interval between two subsequent contractions and the delta (latency; the time interval between stimulus and response) were measured.

### Statistical Analysis

All statistical analyses were performed using Statistical Package for the Social Sciences (SPSS, ver: 19.0), and the comparisons of the means of the physiological parameters were conducted using non-parametric Kruskal Wallis and Mann-Whitney tests. The statistical significance was considered as P<0.05. 

## Results

### Muscle Stimulation

Table 1 shows the pulse height of contractions, the time interval between two subsequent contractions and the latency period in non-exposed control group after in-vivo sham-exposure, and when isolated gastrocnemius muscles were sham-exposed to mobile jammer radiation. As it was expected, no statistically significant differences were observed in different sham exposure phases.

**Table 1:** The pulse height of contractions, the time interval between two subsequent contractions and the latency period in non-exposed Mobile Phone Jammers group after in-vivo sham-exposure, and when isolated gastrocnemius muscles were sham-exposed to mobile phone Jammers (muscle stimulation).

**Control**	**Sample size**	**Phase I**	**Phase II**	**Phase III**	**Phase IV**	**p-value**
		**After in vivo** **sham-exposure**	**After 10 min ** **sham-exposure**	**After 10 min off**	**After 10 min ** **sham-exposure**	
Pulse height of contraction(mV)	10	0.324 ± 0.180	0.295 ± 0.163	0.316 ± 0.147	0.304 ± 0.140	0.68
Time interval(sec)	10	1 ± 0.001	1 ± 0.0008	0.999 ± 0.001	1 ± 0.001	0.083
Latency period(sec)	10	0.004 ± 0.001	0.004 ± 0.002	0.004 ± 0.001	0.004 ± 0.001	0.79

The pulse height of contractions, the time interval between two subsequent contractions and the latency period in moboile phone jammer group after in-vivo exposure, and when isolated gastrocnemius muscles were exposed to mobile jammer radiation are summarized in Table 2.  We observed a statistically significant difference among the latency periods in different off/on exposure phases (P=0.0001). The mean (±SD) latency period after a 2 hour in-vivo exposure was 0.006 ± 0.001 seconds but when isolated gastrocnemius muscles were in 10 min on/off/on exposure phases, the latencies were 0.009 ± 0.001, 0.004 ± 0.001, 0.008 ± 0.001 seconds, respectively.

**Table 2:** **Panel A:** The pulse height of contractions, the time interval between two subsequent contractions and the latency period in jammer group after in-vivo exposure, and when isolated gastrocnemius muscles were exposed to Mobile Phone Jammers (muscle stimulation). **Panel B:** Pairwise comparisons of the latency period.

**Panel A**									
**Jammer**	**Sample size**	**Phase I**		**Phase II**		**Phase III**	**Phase IV**	**p-value**
		**After in vivo** **exposure**		**After 10 min** **exposure**		**After 10 min** **off**	**After 10 min ** **exposure**	
Magnitude of contraction(mV)	10	0.407 ± 0.208		0.446 ± 0.202		0.473 ± 0.166	0.443± 0.157	0.54
Time interval(sec)	10	1 ± 0.0007		1 ± 0.0009		1 ± 0.0008	1 ± 0.001	0.67
Latency period(sec)	10	0.006 ± 0.001		0.009 ± 0.001		0.004 ± 0.001	0.0077 ± 0.001	0.0001
**Panel B**									
**Latency period**	**Phase I**	Phase II		0.02			
		Phase III		0.20			
		Phase IV		0.053			
	**Phase II**	Phase III		0.03			
		Phase IV		0.15			
	**Phase III**	Phase IV		0.03			

**Figure 1:**
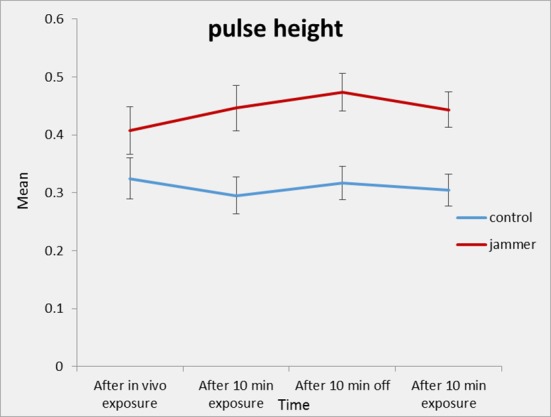
Pulse height of contraction (ph) – Muscle Stimulation (Error bars indicate SE)

**Figure 2:**
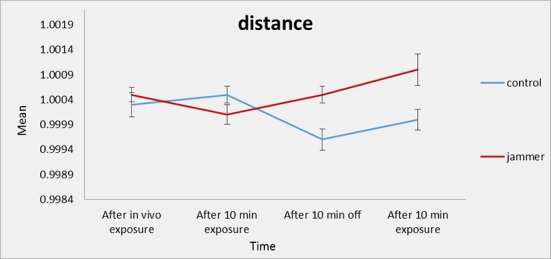
The time interval between two subsequent contractions (distance) – Muscle Stimulation (Error bars indicate SE)

**Figure 3:**
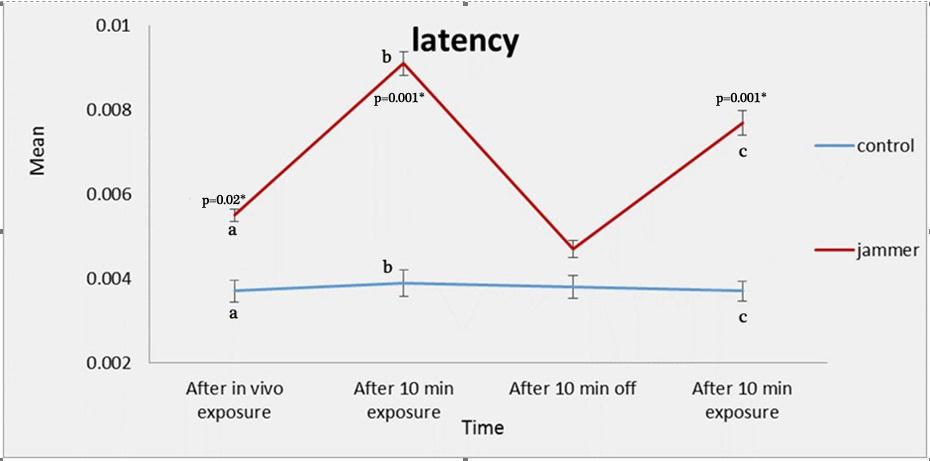
The latency period – Muscle Stimulation (Error bars indicate SE)

The inter-group comparison could not show any statistically significant differences in pulse height and time interval between two subsequent contractions (Figure 1-2).However, a statically significant difference was found in the latency (Figure 3).

### Nerve Stimulation

Table 3 shows the pulse height of contractions, the time interval between two subsequent contractions and the latency period in non-exposed control group after in-vivo sham-exposure, and when isolated gastrocnemius muscles were sham-exposed to mobile jammer radiation. In contrast with what was expected, a statistically significant difference was observed for the pulse height of contractions in different sham exposure phases (P=0.033).

**Table 3:** **Panel A:** The pulse height of contractions, the time interval between two subsequent contractions and the latency period in non-exposed control group after in-vivo sham-exposure, and when isolated gastrocnemius muscles were sham-exposed to Mobile Phone Jammers (Nerve stimulation). **Panel B:** Pairwise comparisons of the pulse height of contractions.

**Panel A**									
**Control**	**Sample size**	**Phase I**		**Phase II**		**Phase III**		**Phase IV**	**p-value**
		**After in vivo** **sham-exposure**		**After 10 min** **sham-exposure**		**After** **10 min off**		**After 10 min** **sham-exposure**	
Pulse height of contraction(mV)	10	0.447 ± 0.18		0.424 ± 0.187		0.368 ± 0.189		0.353 ± 0.208	0.033
Time interval(sec)	10	0.999 ± 0.001		1 ± 0.001		1 ± 0.001		1 ± 0.001	0.69
Latency period(sec)	10	0.004 ± 0.001		0.005 ± 0.001		0.005 ± 0.001		0.005 ± 0.001	0.093
**Panel B**									
**Pulse height of contraction**	**Phase I**	Phase II		0.88		
		Phase III		0.12		
		Phase IV		0.12		
	**Phase II**	Phase III		0.09		
		Phase IV		0.04		
	**Phase III**	Phase IV		0.39		

**Figure 4:**
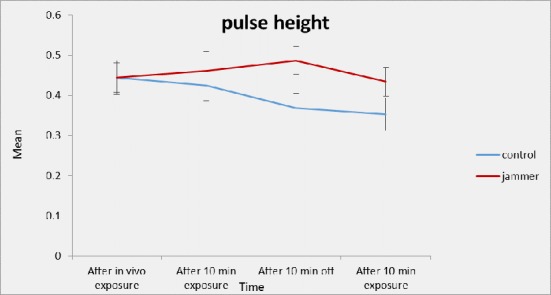
Pulse height of contraction (ph) – Nerve Stimulation (Error bars indicate SE)

**Figure 5:**
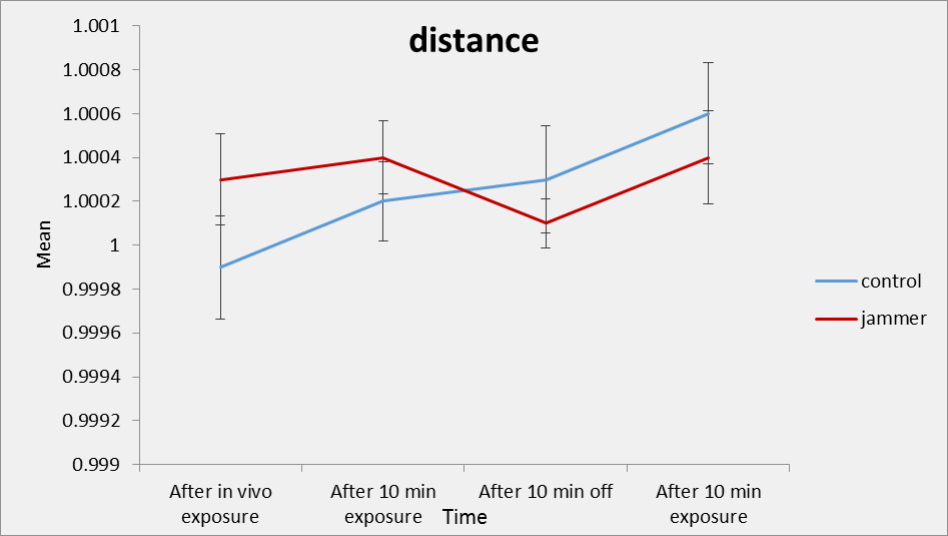
The time interval between two subsequent contractions – Nerve Stimulation (Error bars indicate SE)

The pulse height of contractions, the time interval between two subsequent contractions and the latency period in mobile jammer group after in-vivo exposure, and when isolated gastrocnemius muscles were exposed to mobile jammer radiation are summarized in Table 4.  In this part of the experiment, we observed only a statistically significant difference among the latency periods in different off/on exposure phases (P=0.0001). The mean (±SD) latency period after a 2 hour in-vivo exposure was 0.006 ± 0.001 seconds but when isolated gastrocnemius muscles were in 10 min on/off/on exposure phases, the latencies were 0.009 ± 0.001, 0.006 ± 0.001, 0.009 ± 0.001  seconds, respectively. The inter-group comparison could not show any statistically significant differences in pulse height and time interval between two subsequent contractions (Figure 4-5).

**Table 4:** **Panel A:** The pulse height of contractions, the time interval between two subsequent contractions and the latency period in jammer group after in-vivo exposure, and when isolated gastrocnemius muscles were exposed to Mobile Phone Jammers (Nerve stimulation). **Panel B:** Pairwise comparisons of the latency period.

** Panel A **									
**Jammer**	**Sample size**	**Phase I**		**Phase II**		**Phase III**	**Phase IV**	**p-value**
		**After in** **vivo exposure**		**After 10 min** **exposure**		**After 10 min** **off**	**After 10 min** **exposure**	
Magnitude of contraction(mV)	10	0.444 ± 0.212		0.460 ± 0.248		0.487± 0.177	0.434 ± 0.183	0.056
Time interval(sec)	10	1 ± 0.001		1 ± 0.001		1 ± 0.0005	1 ± 0.001	0.72
Latency period(sec)	10	0.006 ± 0.001		0.009 ± 0.001		0.006 ± 0.001	0.009 ± 0.001	0.0001
**Panel B**									
**Latency period**	**Phase I**	Phase II		0.02			
		Phase III		0.17			
		Phase IV		0.02			
	**Phase II**	Phase III		0.04			
		Phase IV		0.86			
	**Phase III**	Phase IV		0.04			

**Figure 6:**
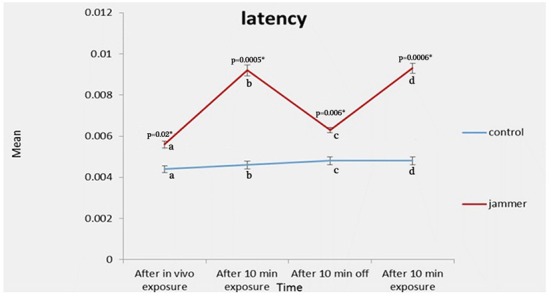
The latency period– Nerve Stimulation (Error bars indicate SE)

However, a statically significant difference was found in the latency (Figure 6).

Table 5 shows the calculated SAR values (W/kg) for 3G, CDMA, DCS and GSM jammer’s antenna.

**Table 5:** Calculated SARs (W/kg).

**Distance**	**3G**		**CDMA**		**DCS**		**GSM**	
	**SAR ** **(W/kg)**		**SAR ** **(W/kg)**		**SAR ** **(W/kg)**		**SAR ** **(W/kg)**	
	**(muscle)**	**(nerve)**	**(muscle)**	**(nerve)**	**(muscle)**	**(nerve)**	**(muscle)**	**(nerve)**
1 meter	0.017	0.010	0.033	0.02	0.038	0.024	0.052	0.033

## Discussion

In this study it is revealed that the pulse height of contractions in frog’s gastrocnemius muscle cannot be affected by the exposure to electromagnetic fields. However, our experiments showed that the latency period can be easily altered by the irradiation. However, none of the experiments could show an alteration in the time interval between two subsequent contractions after exposure to electromagnetic fields. The general pattern which could be observed in these experiments indicates that exposure to electromagnetic fields usually increase the latency period. To the best of our knowledge, this is the first report that shows the role of exposure to electromagnetic fields generated by mobile phone jammers on the pulse height of contractions, time interval between two subsequent contractions and the latency period in frog’s gastrocnemius muscle after applying single square pulses of 1V (1 Hz) as stimuli.

Generally speaking our results are in line with numerous reports which showed that a wide variety of cells including epithelial, endothelial and epidermal cells, cardiac muscle cells, fibroblasts, yeast, E. coli, developing chick eggs, and dipteran cells respond to EMF, both in vivo and in vitro [16]. It has been reported that tissue cultured cells are less susceptible to the effects of EMF, possibly due to this point that immortalized cells have been altered in an important manner to enable them to live limitlessly in unnatural laboratory conditions. However, our results are in contrast with some old reports which indicated that exposure of the isolated frog sciatic nerves, cat saphenous nerves, rabbit vagus nerves, superior cervical ganglia, and rat diaphragm muscles to 2450 MHz microwave radiation cannot change the characteristics of nerves or muscles exposed to CW specific absorption rate (SAR) of 0.3-1500 W/kg and pulsed peak SAR of 0.3-220 kW/kg [17].

Our findings support early reports which indicated the non-thermal effects of electromagnetic radiation on amphibians [18, 19]. Some early reports have indicated that exposure of amphibians to electromagnetic fields may cause teratogenic effects [20]. On the other hand, it is reported that common frogs (Rana temporaria L.) developed under electromagnetic field shows a higher rate of mortality, slow and less synchronously development, allergies, and alterations in their blood counts [21].

It has been also reported that exposure of frogs to electromagnetic radiation with power densities of 30–60 mWcm-2 caused a heart rhythm change, possibly due to activation of the nervous system. Furthermore, an increase in the heart rate and arrhythmia after irradiation of toad hearts with pulses of 1425 MHz at a power density of 0.6 mWcm-2, has been reported [18]. Interestingly, in Spain A. Balmori in a paper entitled “The incidence of electromagnetic pollution on the amphibian decline: Is this an important piece of the puzzle?” published in the journal Toxicological and Environmental Chemistry in 2006 reports the possible association of exposure to microwave radiation and the global disappearance of frogs [22].
